# Ferroptosis-mediated Crosstalk in the Tumor Microenvironment Implicated in Cancer Progression and Therapy

**DOI:** 10.3389/fcell.2021.739392

**Published:** 2021-11-02

**Authors:** Yini Liu, Chunyan Duan, Rongyang Dai, Yi Zeng

**Affiliations:** Department of Biochemistry and Molecular Biology, Southwest Medical University, Luzhou, China

**Keywords:** ferroptosis, metabolism, cancer progress, immunity, tumor microenvironment

## Abstract

Ferroptosis is a recently recognized form of non-apoptotic regulated cell death and usually driven by iron-dependent lipid peroxidation and has arisen to play a significant role in cancer biology. Distinct from other types of cell death in morphology, genetics, and biochemistry, ferroptosis is characterized by the accumulation of lipid peroxides and lethal reactive oxygen species controlled by integrated oxidant and antioxidant systems. Increasing evidence indicates that a variety of biological processes, including amino acid, iron, lactate, and lipid metabolism, as well as glutathione, phospholipids, NADPH, and coenzyme Q10 biosynthesis, are closely related to ferroptosis sensitivity. Abnormal ferroptotic response may modulate cancer progression by reprogramming the tumor microenvironment (TME). The TME is widely associated with tumor occurrence because it is the carrier of tumor cells, which interacts with surrounding cells through the circulatory and the lymphatic system, thus influencing the development and progression of cancer. Furthermore, the metabolism processes play roles in maintaining the homeostasis and evolution of the TME. Here, this review focuses on the ferroptosis-mediated crosstalk in the TME, as well as discussing the novel therapeutic strategies for cancer treatment.

## Introduction

Iron is the most abundant element, by mass, in the Earth (Frey and Reed, 2012), and it is required in a variety of important biological processes in the human body, such as oxygen transport, DNA biosynthesis, adenosine triphosphate (ATP) synthesis, etc. (Bogdan et al., 2016). However, recent studies have increasingly found that disruption of iron metabolism and attendant overload of iron were closely related to the occurrence and development of tumors (Bogdan et al., 2016; Manz et al., 2016). In addition, the presence of iron, especially divalent iron, greatly accelerates lipid peroxidation of saturated fatty acids (FAs) in humans (Pratt et al., 2011). During the iron-related oxidative phosphorylation process in mitochondria, cells produce reactive oxygen species (ROS) along with the generation of ATP. ROS levels that exceed the antioxidation capacity of cells can lead to an oxidative stress response, which directly or indirectly damages proteins, nucleic acids, lipids, and other large molecular substances (Ray et al., 2012), leading to cell injury or death. This newly discovered iron-dependent form of regulated cell death (RCD) caused by unrestricted lipid peroxidation-mediated membrane damage is defined as ferroptosis (Dixon et al., 2012).

Cell death, which is currently divided into accidental cell death (ACD) and RCD (Galluzzi et al., 2018), is closely linked with oxidative stress and tightly integrated with diverse aspects of biological processes in various organisms. Unlike ACD (an uncontrolled and unavoidable process), RCD can be modulated by pharmacological or genetic interventions based on a series of specific intrinsic cellular mechanisms and signaling pathways. Recently, RCD has been classified into apoptotic or non-apoptotic forms (ferroptosis (Dixon et al., 2012), necroptosis (Weinlich et al., 2017), pyroptosis (Bergsbaken et al., 2009), and alkaliptosis (Song et al., 2018b; Liu et al., 2020)), with distinct features in terms of the molecular mechanism and the signals transduction as well as disease implications. Ferroptosis, one such newly identified non-apoptotic modality of cell death, is obviously distinct from other types of RCD in cell morphology, biochemistry, and genetics. Morphologically, cells undergoing ferroptosis usually have the typical necrosis-like morphological changes, such as swelling of the cytoplasm organelles and plasma membrane rupture. These features are distinct from traditional apoptotic cells that are characterized by cell plasma membrane blebbing and shrinking, fragmentation and margination of chromatin, generation of apoptotic bodies, and cytoskeleton breakdown. Biochemically, it is becoming clear that ferroptosis is primarily triggered by the massive lipid peroxidation-mediated membrane rupture in an iron-dependent manner and modulated by lipid repair systems involving glutathione (GSH) and phospholipid peroxidase glutathione peroxidase 4 (GPX4) (Yang et al., 2014). Genetically, a few genes involved in lipid metabolism, such as prostaglandin-endoperoxide synthase 2 (PTGS2/COX2) (Yang et al., 2014), Acyl-CoA synthetase long-chain family member 4 (ACSL4) (Yuan et al., 2016b; Doll et al., 2017), as well as genes responsible for antioxidant defense, have been considered as biomarkers of ferroptosis. As a kind of selenoprotein, active GPX4 can covert potentially toxic lipid hydroperoxides (L-OOH) to non-toxic lipid alcohols (L-OH) (Ursini et al., 1982; Friedmann Angeli et al., 2014; Yang et al., 2014), and suppress the activation of arachidonic acid (AA)-metabolizing enzymes (Kagan et al., 2017).

Mounting evidence has shown that a great variety of human diseases related to iron or ROS including cancer have been connected to the malfunctions of ferroptosis (Stockwell et al., 2017; Galluzzi et al., 2018; Fang et al., 2019). Of note, ferroptosis has been reported to play a dual role in tumor promotion and suppression in different models. As previously mentioned, iron is an essential element for cell proliferation and a co-factor for metabolic enzymes. The absorption of iron by cancer cells can promote the occurrence and progression of tumors. Furthermore, ferroptotic cells may contribute to tumor initiation by playing a positive role in inflammation through immunogenicity within the tumor microenvironment (TME). In contrast, the ferroptosis inducers, such as FDA-approved anti-cancer drugs sorafenib, sulfasalazine, and artesunate, have shown a strong ability to kill cancer cells. Meanwhile, ferroptosis could also mediate the tumor suppression activity of interferon γ (IFNγ) released by CD8^+^ T cells (Stockwell and Jiang, 2019). In particular, cancer cells with malignant mutations that are resistant to common cancer therapies or with a mesenchymal-like phenotype might be vulnerable to ferroptosis (Hangauer et al., 2017; Viswanathan et al., 2017). Therefore, in addition to the effects of oncogenes and tumor suppressors, the TME also plays a critical role in the complex process of ferroptosis in tumor treatment and tumorigenesis.

The TME refers to the complex and diverse multicellular environment in which tumor cells develop. In general, the TME comprises the peripheral blood and lymphatic vessels, immune cells, stromal cells, bone marrow-derived inflammatory cells, extracellular matrix (ECM), and various secreted signaling molecules (Quail and Joyce, 2013). Tumors and TME are often referred to as the relationship between “seed” and “soil,” and the TME plays an essential role in tumor growth and progression. Notably, cancer cells can affect the microenvironment by releasing cell signaling molecules to promote tumor angiogenesis and induce immune tolerance, while immune cells in the microenvironment could regulate the growth and development of cancer cells. Emerging evidence has shown that ferroptosis is closely related to cancer progression and therapy. The triggers of ferroptosis, including iron accumulation, lipid peroxidation, and ROS, are generated by a series of environmental and genetic stimuli, such as heat and radiation exposure, metabolism, redox homeostasis, and intercellular interactions, as well as oncogenic and tumor-suppressive signaling (Ryter et al., 2007; Berghe et al., 2014). On the other hand, recent evidence indicated that ferroptotic cancer cells could release signal molecules, including oxidized lipid mediators, eicosanoids, and high mobility group box1 (HMGB1) (Friedmann Angeli et al., 2014; Kagan et al., 2017; Wen et al., 2019), into the ECM to modulate the anti-cancer immunity. These findings suggested the potential roles of TME in regulating ferroptosis, and the ferroptosis of cancer cells could also affect the TME in turn.

Recently, the process and function of ferroptosis and its impact on disease have been better explained. Although ferroptosis plays an important role in maintaining the survival of normal cells and tissues, it is increasingly recognized that some carcinogenic pathways are related to ferroptosis, which makes cancer cells vulnerable to ferroptosis and death (Yu et al., 2017; Wang Y. et al., 2020). Here, we aimed to discuss the emerging regulatory network between ferroptosis and TME in cancer cells and its potential role in cancer treatment.

## The Discovery of Ferroptosis

The early observation of ferroptosis-like cell death can be traced back to the mid-twentieth century, when oxidative stress-induced cell death was detected in neuronal cells and metabolism studies termed “oxytosis” (Tan et al., 2001). In the 1950s and 1960s, Harry Eagle’s pioneering work demonstrated a ferroptosis-like cell death that is induced by the lack of the amino acid cysteine (Eagle, 1955), and the endogenous synthesis of cysteine makes cells resistant to this cell death (Coltorti et al., 1956; Eagle et al., 1961). The first identified ferroptosis inducer was caught in the search for new therapeutic drugs targeting RAS mutation, which is often involved in cancer. In 2003, Dolma et al. identified a small chemical compound, erastin, by using synthetic lethal high-throughput screening, which can cause selectively toxic to cancer cells expressing oncogenic RAS, but not wild-type cells in a manner different from traditional apoptosis (Dolma et al., 2003). Subsequently, RAS-selective lethal small molecule (RSL)3 and RSL5, which have the same effect as erastin that leads to a nonapoptotic, MEK-dependent, and iron-dependent oxidative cell death, were discovered in 2008 (Yang and Stockwell, 2008). In 2012, Dixon et al. formally coined this kind of RCD to ferroptosis based on the characteristics that the anti-cancer activity of erastin in RAS-mutated tumors could be completely blocked by the iron chelators and lipophilic antioxidants (instead of the apoptotic inhibitor Z-VAD-FMK) (Dixon et al., 2012). The term “ferroptosis” is a combination of the Greek word “ptosis” for corruption and the Latin word “Ferrum” for iron, indicating the importance of iron in this non-apoptotic form of cell death (Tsoi et al., 2018). Afterward, multiple inducers of ferroptosis, such as sorafenib (Louandre et al., 2013), artemisinins (Eling et al., 2015; Ooko et al., 2015), FIN56 (Liang et al., 2019), and FINO_2_ (Abrams et al., 2016), were identified to affect the accumulation of lipid peroxidation products and lethal ROS derived from iron metabolism. Although ferroptosis has only been observed in mammalian systems in recent years (Dixon et al., 2012), it is considered to be one of the most widespread and oldest forms of cell death, since ferroptotic-like cell death has also been observed in evolutionary-distant species, such as those belonging to plants, protozoa, and fungi (Distefano et al., 2017; Bogacz and Krauth-Siegel, 2018; Shen et al., 2020). Since the Nomenclature Committee on Cell Death (NCCD) named such RCD type as “ferroptosis”, it gradually entered the explosive stage of ferroptosis research, and provided a novel insight into cancer research.

## The Regulatory Mechanisms of Ferroptosis

Ferroptosis is a ROS-dependent form of cell death associated with the accumulation of excessive iron and lipid peroxidation, as well as changes in specific genes involved in regulating iron homeostasis and lipid peroxidation metabolism (Yan et al., 2021). Generally, increased free iron and lipid peroxidation are considered to be the two key components that contribute to oxidative damage to the lipid bilayer of the membrane in ferroptosis (Dixon et al., 2012). The process of ferroptosis is regulated by the balance between integrated oxidation and antioxidant systems (Chen et al., 2021).

### The Activation Mechanisms of Ferroptosis

Lipid peroxidation is a trigger of ferroptosis and is caused by a complicated process of lipid metabolism. In the process of ferroptosis, polyunsaturated fatty acids (PUFAs), especially AA and adrenic acid (AdA), are sensitive to be oxidized to lipid peroxides by ROS, resulting in damage to the lipid bilayer and thus promoting ferroptosis. ACSL4 and lysophosphatidylcholine acyltransferase 3 (LPCAT3) are required for biosynthesis and modification of PUFAs in cell membranes and act as important drivers of ferroptosis. In the ferroptotic process, ACSL4 catalyzes the ligation of AA and AdA with coenzyme A to form lipid derivatives AA-CoA or AdA-CoA (Yuan et al., 2016b; Doll et al., 2017; Kagan et al., 2017). These products can be re-esterified into membrane phosphatidylethanolamine (PE) by LPCAT3 to produce AA-PE or AdA-PE (Dixon et al., 2015; Doll et al., 2017). However, loss function of ACLS4 could induce a substantial change of long-chain PUFAs tails to short-chain and monounsaturated fatty acids (MUFAs) tails in phospholipids (Kim et al., 2001; Doll et al., 2017). These MUFAs, including exogenously supplemented and endogenously produced stearoyl-CoA desaturase 1 (SCD1), can be converted to acyl-coenzyme A esters, which can bind to the membrane phospholipids, thereby protecting cancer cells from ferroptotic cell death (Chen et al., 2021). Thus, depletion of ACSL4 or LPCAT3 could inhibit ferroptosis by reducing the accumulation of lipid peroxidation substrates in cells.

Moreover, the accumulation of iron can promote the activity of arachidonate lipoxygenases (ALOXs), which are a class of non-heme iron-dependent enzymes, and induce ferroptosis. ALOXs, including ALOXE3, ALOX5, ALOX12, ALOX12B, ALOX15, and ALOX15B, can directly oxygenate PUFAs to generate various hydroperoxy PUFA derivatives, such as lipid hydroperoxides (LOOHs), malondialdehyde (MDA), and 4-hydroxynonenal (4HNE), thus inducing ferroptosis (Kagan et al., 2017). Moreover, ALOXs also play important physiological roles in the immune system by producing pro-inflammatory and anti-inflammatory molecules that directly modulate (neuro) inflammatory processes and the TME (Friedmann Angeli et al., 2019; Proneth and Conrad, 2019). Besides the context-dependent role of ALOXs in ferroptosis, recent findings suggest that the cytochrome P450 oxidoreductase (POR) also plays a role in promoting lipid peroxidation in cancer cells in an ALOX-independent manner (Zou et al., 2020). Prostaglandin-endoperoxide synthase 2 (PTGS2/COX2) is not involved in the oxidation of phospholipids, but can oxidize lysophospholipids. PTGS2 is usually upregulated during ferroptosis and considered as a biomarker, rather than as a driver, of ferroptosis (Yang et al., 2014). However, the inhibitor of PTGS has little effect on the lethality of erastin or RSL3, but PTGS2 may modulate ferroptosis in neural cells after traumatic brain injury (Yang et al., 2014; Yang et al., 2016; Xiao et al., 2019). The specific role of PTGS2 in ferroptosis of cancer cells remains to be elucidated.

### The Inhibition Mechanisms of Ferroptosis

The antioxidant enzyme GPX4 can covert GSH into oxidized glutathione (GSSG) and directly reduce toxic phospholipid hydroperoxides (PL-OOH) to nontoxic phospholipid alcohols (PLOH), thus acting as a central repressor of ferroptosis in cancer cells (Yang et al., 2014). Inactivation of GPX4-dependent antioxidant defense leads to the accumulation of the lipid peroxides, which is a trigger of ferroptosis. Selenium and GSH are necessary components for the expression and activity of GPX4 in ferroptosis (Ingold et al., 2018; Ursini and Maiorino, 2020). Selenium can boost the anti-ferroptosis activity of GPX4 by inserting selenocysteine into GPX4 residue at Sec 46 (U46) (Ingold et al., 2018). Meanwhile, selenium-supplementation has been demonstrated to upregulate GPX4 expression by coordinated activation of the transcription factor AP-2 gamma (TFAP2C) and specificity protein 1 (SP1), which protects against ferroptosis-related cerebral hemorrhage (Alim et al., 2019). The antioxidant GSH is composed of three amino acids, cysteine, glycine, and glutamic acid; cysteine is considered to be the main rate-limiting factor in *de novo* synthesis of GSH. In mammalian cells, cystine (the oxidized form of cysteine) is imported into cells by system Xc^−^ for subsequent GLC (glutamate-cysteine ligase)-mediated GSH production. As a transporter that facilitates the exchange of cystine and glutamate across the plasma membrane, system Xc^−^ is a heterodimeric protein complex consists of two subunits: solute carrier family 7 member 11(SLC7A11; also known as xCT) and solute carrier family 3 member 2 (SLC3A2, also known as CD98 or 4F2). The expression of SLC7A11 is promoted by nuclear factor erythroid 2 like 2 (NFE2L2/NRF2) and downregulated by the tumor suppressor genes, such as TP53, BAP1, and BECN1 (Jiang et al., 2015; Song et al., 2018a; Zhang et al., 2018). This dual regulation of SLC7A11 expression and activity creates a fine-tuning mechanism to modulate cell antioxidant capacity and lipid ROS during ferroptosis (Chen et al., 2021). Inhibition system Xc^−^ (with erastin, sulfasalazine, or sorafenib) or GPX4 (with RSL3, ML162, ML210, FIN56, or FINO2) can produce ROS in the absence of GSH depletion and trigger ferroptosis. Similarly, depletion of SLC7A11 or GPX4 promotes lipoxygenase-mediated lipid peroxidation and leads to ferroptosis in specific cells or organs (Sato et al., 2005; Friedmann Angeli et al., 2014). In addition, the endogenous antioxidant coenzyme Q10 (CoQ10) produced by the mevalonate (MVA) pathway is converted to a reduced form to suppress lipid peroxidation, thereby protecting cells from undergoing ferroptosis in a GPX4-independent manner (Mullen et al., 2016). Tetrahydrobiopterin (BH_4_) and ESCRT (endosomal sorting complexes required for transport)-III membrane repair systems also show a context-dependent antioxidant property (independent of the Cysteine/GSH/GPX4 axis) during ferroptosis (Dai et al., 2020c; Kraft et al., 2020). Furthermore, the ferroptosis suppressor protein 1 (FSP1/AIFM2) controls the generation of reduced CoQ10, and can also inhibit ferroptosis in cancer cells by activating the ESCRT-III membrane repair system (Dai et al., 2020d). This indicates that the antioxidant system could prevent ferroptosis through mutual synergy or complementation.

## Role of Ferroptosis in Cancers

As previously mentioned, the earliest chemical inducer of ferroptosis was discovered in screening for novel therapeutic compounds targeting cancers with RAS mutation (Dolma et al., 2003). Subsequent studies have revealed that excessive or defective ferroptosis is tightly implicated in the treatment and tumorigenesis of various tumor types. In general, cancer cells are thought to be more susceptible to ferroptosis due to their active metabolism, high ROS load, and high iron supply demand. The sensitivity of different tumor types to ferroptosis, on the other hand, varies, which may be due to a distinct genetic background or epigenetic modification. In this section, we summarize the role of ferroptosis in various tumors.

### Pancreatic Cancer

Pancreatic ductal adenocarcinoma (PDAC) is a one of the deadliest tumors that is resistant to most therapies. In recent years, an increasing number of researchers have investigated the role of ferroptosis in the treatment, initiation, and progression of pancreatic cancer. Daniel et al. found that cytosolic aspartate aminotransaminase (GOT1) inhibition represses mitochondrial metabolism and enhances labile iron availability through autophagy, which accelerates pancreatic cancer cell death by ferroptosis (Kremer et al., 2021). Furthermore, specific and conditional depletion of pancreatic *Slc7a11* could induce pancreatic tumor ferroptosis and inhibit pancreatic tumorigenesis in mice (Badgley et al., 2020). However, ferroptosis may play oncogenic roles in pancreatic cancer in addition to suppressing tumor progression. During *Kras*-driven PDAC in mice, ferroptotic cancer cells promote the release of damage-associated molecular patterns (DAMPs) such as 8-hydroxy-2ʹ-deoxyguanosine (8-OHG) and KRAS-G12D protein, leading to macrophage polarization and subsequent pancreatic tumor growth (Dai et al., 2020a; Dai et al., 2020b). The dual role of ferroptosis in pancreatic cancer needs to be considered when developing new cancer therapeutic approaches based on ferroptosis induction.

### Hepatocellular Carcinoma (HCC)

HCC is the most major form of liver cancer diagnoses and deaths (McGlynn et al., 2021). Clinically, sorafenib is one of the six approved systemic therapies for patients with advanced HCC (Llovet et al., 2021), and inducing ferroptosis of cells is an important mechanism in sorafenib treating HCC. In terms of mechanism, sorafenib blocks the import of cysteine by inhibiting SLC7A11, the subunit of cystine/glutamate antiporter system Xc^−^, thereby triggering ferroptosis and ultimately killing HCC cells (Louandre et al., 2013). The effect of sorafenib on HCC cells is closely related to ferroptosis process. GSH depletion through cysteine deprivation or cysteinase inhibition can enhance the susceptibility of HCC cells to sorafenib-induced ferroptosis, which provides a new sight in therapeutic combinations for advanced HCC (Li Y. et al., 2021). Moreover, research also reported that iron chelator deferoxamine (DFX) protects HCC cells from the sorafenib-induced ferroptotic cell death (Louandre et al., 2013). The status of retinoblastoma (Rb) protein modulates the tumorigenesis of liver and the response of human liver cancer cells to sorafenib (Mayhew et al., 2007; Louandre et al., 2015). HCC cells with Rb-negative status promote the occurrence of ferroptosis and cell death upon exposure to sorafenib (Louandre et al., 2015).

It has been reported that several proteins, such as NRF2, DAZAP1, metallothionein-1G (MT-1G), Branched-chain amino acid aminotransferase 2 (BCAT2), and CDGSH iron-sulfur domain 1 (CISD1), inhibit ferroptosis in HCC cells through different ways. Sun et al. revealed that activation of the p62-Keap1-NRF2 pathway prevents HCC cells from ferroptosis, and the Ras/Raf/MEK pathway is an important target for ferroptosis in the treatment of HCC (Sun et al., 2016b; Nie et al., 2018). RNA-binding protein DAZAP1 maintains the SLC7A11 mRNA stability to negatively regulate ferroptosis in HCC cells (Wang Q. et al., 2021). MT-1G knockdown increases GSH depletion and lipid peroxidation, thereby enhancing sorafenib-induced ferroptosis (Sun et al., 2016a). The aminotransferase BCAT2 protects HCC cells from system Xc^−^ inhibitor-induced ferroptosis by mediating the metabolism of sulfur amino acid (Wang K. et al., 2021). Inhibition of CISD1, an iron-containing outer mitochondrial membrane protein, contributes to erastin-induced ferroptosis in HCC cells (Yuan et al., 2016a). In addition, inhibition of sigma 1 receptor (S1R) by haloperidol also promotes erastin- and sorafenib-induced ferroptosis in HCC cells (Bai et al., 2017).

### Breast Cancer

Breast cancer is one of the most common causes of cancer-related death in women (DeSantis et al., 2019). The triple-negative breast cancer (TNBC) is an aggressive breast cancer subtype with poor prognosis, and lacks effective therapeutic target. Notably, TNBC have been shown to more sensitive to ferroptosis than estrogen receptor (ER) positive breast cancer, suggesting that ferroptosis is a promising treatment for TNBC patients. The drug-tolerant persister breast cancer cells acquire a dependency on GPX4, making cells sensitive to ferroptosis triggered by GPX4 inhibition (Hangauer et al., 2017). Sulfasalazine (SAS) can trigger ferroptosis in breast cancer cells by inactivating GPX4 and system Xc^−^, especially in cells with low ER expression (Yu et al., 2019). It was observed that the tyrosine kinase inhibitors lapatinib and neratinib induce ferroptosis by increasing the level of iron-dependent ROS in breast cancer cells (Ma et al., 2017; Nagpal et al., 2019). Cystine is one of the most essential amino acids in TNBC. Starvation of cystine reduces the level GSH by directly decreasing synthesis and the GCN2-eIF2α-ATF4-CHAC1 pathway, thereby inducing ferroptosis in TNBC cells (Chen et al., 2017). The anti-diabetic drug metformin reduces the stability of xCT by inhibiting its UFMylation, thus inducing ferroptosis and inhibiting the proliferation of breast cancer cells (Yang et al., 2021). In contrast, MUC1-C, a transmembrane protein, can maintain the GSH level and redox balance by forming a complex with xCT and the CD44 variants (CD44v), which inhibits ferroptosis in breast cancer cells (Ishimoto et al., 2011). Another study found that prominin-2 protects breast cancer cells from ferroptotic cell death by promoting the formation of ferritin-containing multivesicular bodies and exosomes that transport ferritin out of the cell (Brown et al., 2019).

### Lung Cancer

Several studies revealed that iron is closely associated with the development of lung cancer, and the disruption of iron homeostasis makes cells more sensitive to ferroptosis (Kuang and Wang, 2019). USP35 is abundant in lung cancer cells and acts as a deubiquitinase to maintain the protein stability of ferroportin, which is a cellular efflux channel for iron (Tang et al., 2021). Depletion of USP35 can promote ferroptotic cell death and sensitivity to cisplatin and paclitaxel chemotherapy in lung cancer cells (Tang et al., 2021). The iron-sulfur cluster biosynthetic enzyme NFS1 is overexpressed in well-differentiated lung adenocarcinomas and protects cells from ferroptosis within high oxygen environment by sustaining the levels of iron-sulfur cofactors (Alvarez et al., 2017). Accumulating evidence showed that system Xc^−^ is increased in several types of cancers, including lung cancer. As a result, suppression of system Xc^−^ by either genetic depletion or pharmacological inhibition to trigger ferroptosis becomes a potential therapy target for lung cancer. Recently, Wang et al. reported that the stem cell factor SOX2 increases the resistance of lung cancer cells to ferroptosis by increasing the expression of SLC7A11. Oxidation of SOX2 at Cys265 can inhibit its transcriptional activity and make lung cancer cells with high SOX2 expression more susceptible to ferroptosis. (Wang X. et al., 2021). In addition to the typical GSH-dependent GPX4 pathway, FSP1 acts as a ferroptosis-resistance factor by reducing CoQ10 to inhibits lipid peroxidation in lung cancer cells (Bersuker et al., 2019).

### Gastric Cancer

Recently, researchers have shown that the PUFA biosynthesis pathway is critical in the ferroptosis of gastric cancer. Gastric cancer cells with high expression of elongation of very long-chain fatty acid protein 5 (ELOVL5) and fatty acid desaturase 1 (FADS1) are more sensitive to ferroptosis, while cells that highly expressed stearoyl-CoA Desaturase 1 (SCD1) exhibit ferroptosis resistance (Wang C. et al., 2020; Lee et al., 2020). Similarly, inhibition of the GSH-GPX4 antioxidation system, which directly reduces lipid hydroperoxides to nontoxic lipid alcohols, can also induce ferroptosis in gastric cancer. Apatinib, an approved anti-angiogenic agent for advanced gastric cancer therapy, can induce lipid peroxidation by inhibiting the expression of GPX4 in gastric cancer cells, and thus leading to ferroptosis (Zhao et al., 2021). In addition, Sirtuins 6 (SIRT6) is significantly expressed in sorafenib-resistant gastric cancer cells. Depletion of SIRT6 inactivates the Keap1-NRF2 pathway and downregulates GPX4 expression, and enhances the sensitivity of gastric cancer cells to sorafenib-induced ferroptosis (Cai et al., 2021). Cysteine dioxygenase 1 (CDO1), on the other hand, regulates erastin-induced ferroptosis in gastric cancer cells by limiting the synthesis of GSH (Hao et al., 2017). Increasing CDO1 activity can competitively absorb cysteine and oxidize it to its sulfinic acid, thereby reducing cellular GSH levels and promoting ferroptosis.

## The Ferroptosis-related Metabolisms Within the Tumor Microenvironment

In recent years, it has been widely appreciated that TME is involved in dynamically controlling cancer development and impacting therapeutic outcomes. In solid tumors, the vascular system is incomplete due to the rapid growth of cells, so the oxygen supply in tumor tissue is insufficient, and the TME is characterized by hypoxia (Albini and Sporn, 2007). In this context, solid tumor cells mainly metabolize energy through anaerobic glycolysis, which leads to the accumulation of lactic acid and thus acidification of the microenvironment (Ferreira, 2010). At the same time, the initiation and progression of the tumor itself will also trigger the immune response of the immune system, resulting in the buildup of inflammatory cells in the region, leading to a significant inflammatory response. Mounting evidence has shown that ferroptosis plays a dual role in cancer promotion and suppression, which is dependent not only on the expression of oncogenes and tumor suppressors but also on the TME. Meanwhile, the release of DAMPs mediated by ferroptosis damage may maintain an inflammatory TME, thereby limiting anti-tumor immunity. However, the specific mechanism of the crosstalk between ferroptotic cancer cells and their microenvironment remains to be clarified. Therefore, it’s necessary to elucidate the interaction between ferroptosis and TME, which might provide a novel anti-cancer treatment (Binnewies et al., 2018). Here, we discuss the major metabolism processes that influence ferroptosis within the TME.

### Interplay Between Iron Metabolism and Ferroptosis in the TME

Iron is a fundamental element that plays a critical role in a variety of vital biological processes *via* distinct oxidation states (from −2 to +6). In biological systems, iron is primarily restricted to ferrous (Fe^2+^), ferric (Fe^3+^), and ferryl (Fe^4+^) states. Due to its flexible oxidation states, iron is usually employed as an electron transporter in crucial biological reactions, such as DNA synthesis and cellular respiration. As previously mentioned, iron metabolism has been identified as one of the pivotal regulators of ferroptosis. Studies have shown that the excess iron overload could result in ferroptosis by accumulating ROS through the Fenton reaction. In this reaction, hydrogen peroxides react with ferrous iron (Fe^2+^) to produce hydroxyl radicals and ferric iron (Fe^3+^) (Winterbourn, 1995). In turn, the generation of Fe^3+^ in the Fenton reaction can be reduced to Fe^2+^ by the superoxide, a by-product of cellular respiration, to undergo further Fenton reaction. This cycle between oxidized and reduced form of iron, known as Haber-Weiss reaction, enables it to produce massive amounts of harmful ROS, which could attack most cellular proteins, lipids, and nucleic acids (Torti et al., 2018). When the GSH-mediated anti-oxidative system is insufficient to eliminate the increasing iron-dependent lipid peroxides, cells were induced into ferroptosis.

Multiple areas of research have found that cancer cells exhibit a distinct iron metabolism from their non-malignant counterparts (Torti and Torti, 2013; Fonseca-Nunes et al., 2014). Cancer cells, especially cancer stem cells, are iron addicted and uptake more iron into cellular to maintain the activity of iron-dependent proteins and enhance tumor growth and metastasis (Ludwig et al., 2015). The intracellular iron metabolism is tightly regulated by iron regulatory proteins (IRP1 and IRP2), which modulate the cellular Fe^2+^ concentrations by regulating iron import, storage, release, and efflux (Andrews and Schmidt, 2007). Of note, cancer cells likely to express different IRPs to promote iron uptake and repress iron export due to the rapid proliferation rate and high metabolic activities that are commonly associated with malignancy. Down-regulation of IRP2 protects cells against ferroptosis, while depletion of FBXL5, an IRP2 negative regulator, sensitizes HT-1080 cells to erastin-induced ferroptosis (Dixon et al., 2012). Moreover, the ferritin heavy subunit (FTH) produced by cancer cells can activate circulating T cells to secret cytokines such as TNF-α and IFNγ, resulting in increased iron retention in macrophages (Meng et al., 2017). Degradation of FTH by lysosomes can accumulate large amounts of iron (Terman and Kurz, 2013). Increasing FTH levels in fibrosarcoma HT1080 cells by inhibiting lysosomal activity or silencing nuclear receptor activator 4 (NCOA4), a cargo receptor that mediates FTH autophagic degradation and iron release, can inhibit ferroptosis (Gao et al., 2016; Hou et al., 2016). Emerging studies, on the other hand, have revealed that iron metabolism in cancer cells is implicated in the complicated nature of tumor-associated macrophages (TAMs) polarization in the TME. In general, the macrophages with high plasticity can be polarized into two forms: the classical M1 (proinflammatory) and the alternative activated M2 (anti-inflammatory) macrophages (Murray, 2017). The M1 macrophages (activated by microbial agents and/or Th1 cytokines) are highly expressed with iron storage protein ferritin and low expressed with iron exporter ferroportin, thus M1 macrophages exhibit iron-accumulating properties (Agoro et al., 2018). However, M2 macrophages (activated by Th2 cytokines IL-4 or IL-13) display the opposite expression profile, with high expression of ferroportin and low expression of ferritin, which enhanced the release of iron and dysregulated iron metabolism in cancer cells (Recalcati et al., 2010). Changes in iron metabolism-related components involving ferritin, ferroportin, and iron levels could stimulate the M1 and M2 polarization (Liang and Ferrara, 2020). Ferroptotic tumor cells induced by excessive iron overload can release 8-OHG, the main product of oxidative DNA damage, to activate the stimulator of interferon genes protein (STING)-mediated DNA sensor pathway in TAMs, which results in TAMs infiltration and M2 polarization, thus enhancing pancreatic carcinogenesis (Dai et al., 2020b).

**FIGURE 1:**
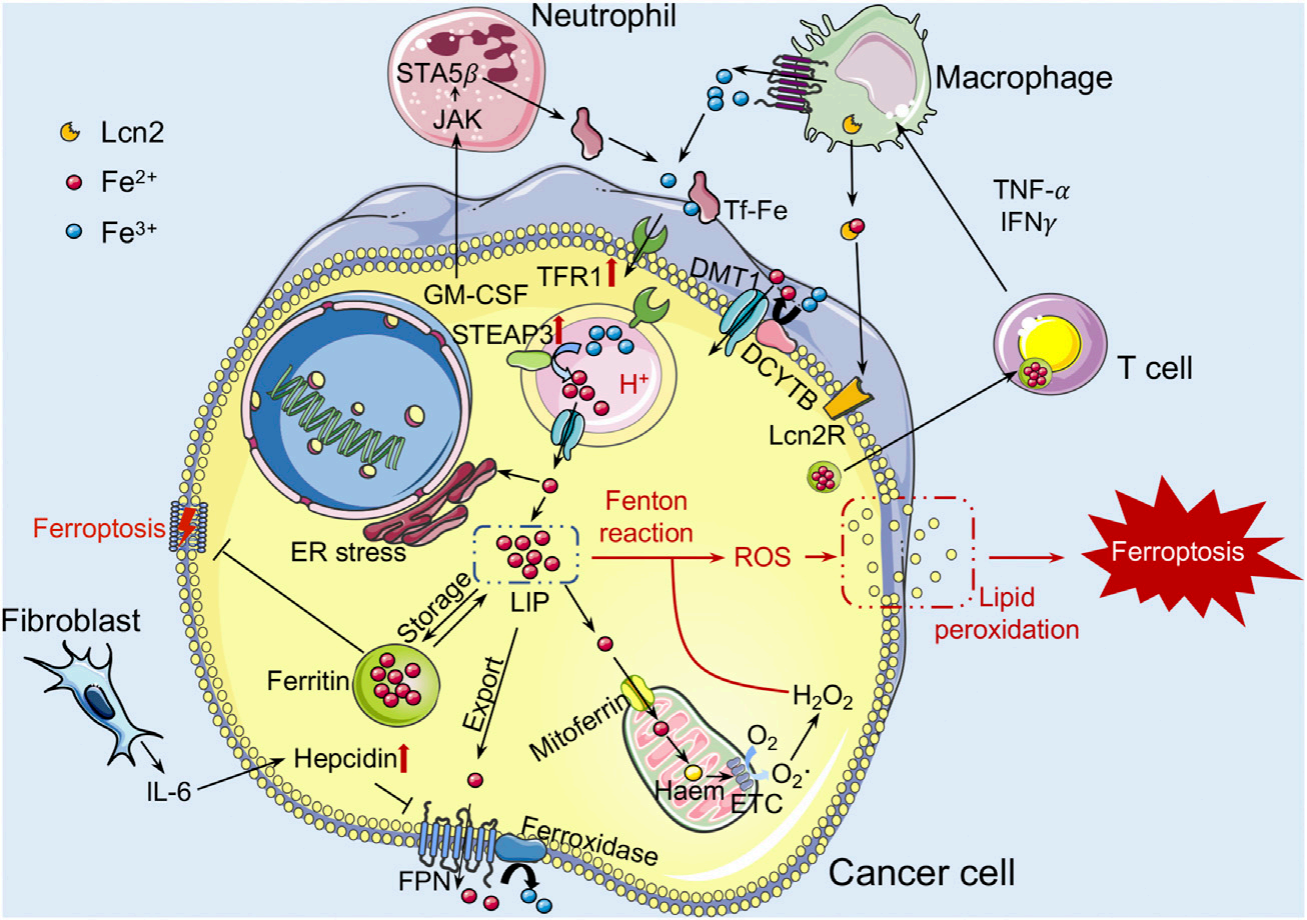
The role of iron metabolism in ferroptosis within the TME. Iron binds to iron-bound transferrin (TF) and enters the cell through endocytosis of transferrin receptor (TFR). Iron is dissociated from the TF-TFR1 complex in the acidified environment of the early endosome, and is transported to the cytoplasm *via* DMT1 after reduced by STEAP3. Next, iron is gathered in the cytoplasm as a labile iron pool (LIP). Iron in the LIP can be stored inside ferritin (Ft) nanocages or transported to the mitochondria to support oxidative respiration. It can also be exported into extracellular to supplement TF-bound iron by ferroxidase binding ferroportin (FPN), which can be blocked by the hepcidin hormone. Increased LIP levels may generate reactive oxygen species (ROS) through Fenton reactions, leading to lipid peroxidation and inducing ferroptosis. TFR1 and STEAP3 proteins tend to be upregulated in cancer cells. In addition, IL-6 produced by CAFs can also increase the production of hepcidin in cancer cells. Cancer cells can also obtain more iron from the M2 macrophages with an iron-releasing phenotype to meet their exuberant metabolic demands. DMT1, divalent metal transport-1; STEAP3, steap3 family member-3; CAFs, cancer-associated fibroblasts; Lcn2, lipocalin 2.

Emerging evidence shows that innate immune cells in the TME, such as TAMs and neutrophils, play a critical role in the regulation of iron metabolism by controlling iron availability in cancer cells (Figure 1). A large amount of iron required for daily iron metabolism was retrieved by macrophages from senescent red blood cells and then returned into the circulation (De Domenico et al., 2008). Therefore, cancer cells are iron consumers, while neutrophils and macrophages are iron providers. Within the TME, TAMs are implicated in cancer cell proliferation, angiogenesis, as well as immunosuppression and therapy resistance (Mantovani et al., 2006; Cassetta and Pollard, 2018). Moreover, TAMs could also increase the release of IRPs, such as the paracrine factor lipocalin 2 (Lcn2), to serve as an iron donor and promote the progression of breast cancer cells (Duan et al., 2018). TAMs can therefore engage in iron metabolism modulation by altering their polarization and interact with cancer cells to influence tumor development. Like macrophages, immune cell neutrophils could also generate iron-related proteins Lcn2 and lactoferrin to affect the iron metabolism in the TME (Cronin et al., 2019). With the stimulation of Lcn2, renal cell carcinoma cells demonstrated resistance to erastin-induced ferroptosis (Meier et al., 2021). In turn, Lcn2 is also involved in promoting the chemotaxis of neutrophils and stimulating M2 polarization to promote tumor progression (Sola et al., 2011; Ye et al., 2016). Hepcidin is an iron-regulatory hormone that induces degradation of ferroportin and negatively regulates iron efflux. Proinflammatory cytokines such as tumor necrosis factor (TNF)-α and IFNγ have been shown to induce hepcidin production in macrophages and neutrophils (Sow et al., 2009; Wu et al., 2012; Blanchette-Farra et al., 2018). Meanwhile, it has been demonstrated that interleukin (IL)-6 generated by cancer-associated fibroblasts (CAFs), TAMs, and neutrophils enhances hepcidin expression, thereby boosting the iron concentration in breast cancer cells (Nemeth et al., 2004; Blanchette-Farra et al., 2018). Overall, the crosstalk between tumor-associated innate immunity in the TME and dysregulated iron metabolism in cancer cells is crucial for triggering ferroptosis.

### Ferroptosis-related Amino Acid Metabolism Linked to the Tumor Immunity in the TME

**FIGURE 2:**
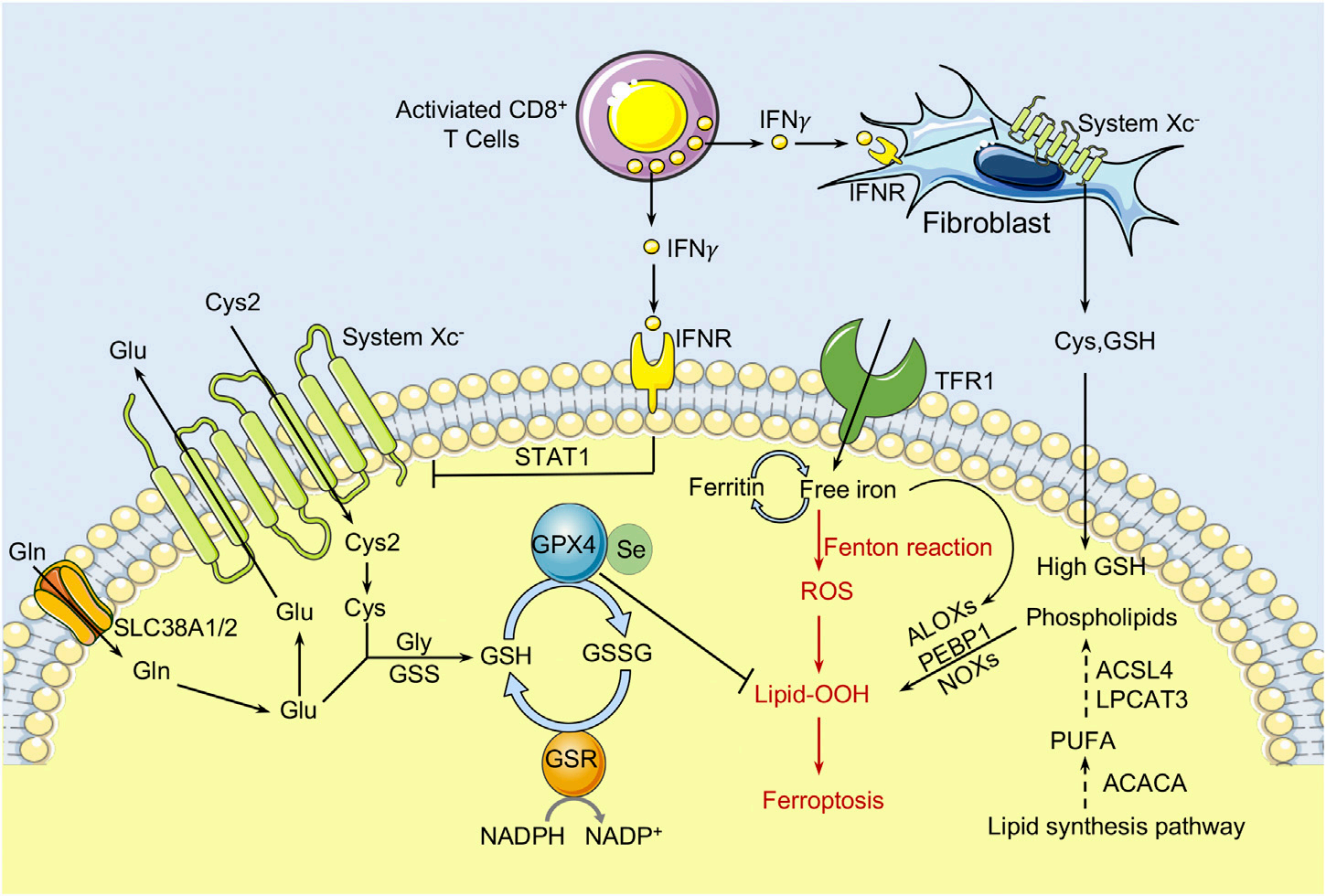
The role of amino acid/glutathione metabolism and lipid metabolism in ferroptosis within the TME. Cystine (Cys2) is transferred into cells by System Xc^−^ at a ratio of 1:1 and then oxidized to cysteine. GPX4 inhibits the toxicity of lipid peroxides through its enzyme activity and maintains the homeostasis of the membrane lipid bilayer. In the process of ferroptosis, PUFAs are prone to peroxidation, resulting in the destruction of the lipid bilayer and affecting membrane function. The free PUFA and CoA are catalyzed by ACSL4 to form derivatives AA-CoA or AdA-CoA, then they are catalyzed by LPCAT3 to form polyunsaturated fatty acid-containing phospholipids (PUFA-PLs). In addition, the immunotreatment-activated CD8^+^ T cells can enhance ferroptosis in cancer cells by releasing IFNγ, which activates the JAK1-STAT1 signaling and thereby inhibit the expression of SLC7A11 and SLC3A2. CAFs could protect cancer cells from chemoresistance-induced oxidative stress by releasing cysteine and GSH to nearby ovarian cancer cells. Meanwhile, CD8^+^ T cells act as a repressor and prevent Cys and GSH release from the CAFs through IFNγ induced SLC7A11 and SLC3A2 downregulation. Cys, cysteine; GPX4, glutathione peroxidase 4; PUFAs, polyunsaturated fatty acids; ACSL4, Acyl-CoA synthetase long-chain family member 4; LPCAT3, lysophosphatidylcholine acyltransferase 3; IFNγ, interferon γ; SLC7A11, solute carrier family 7 member 11; SLC3A2, solute carrier family 3 member 2.

Abnormal iron overload in cancer cells promotes the production of ROS *via* the Fenton reaction and hence increases oxidative stress. Accumulating ROS oxidizes the PUFAs to lipid peroxides to induce ferroptosis of cancer cells. Amino acid metabolism is involved in the regulation of ferroptosis (Figure 2). To protect from ferroptosis, cancer cells activate their antioxidant capabilities through a variety of mechanisms such as increasing demand for cysteine to synthesize the antioxidant GSH. GSH is a tripeptide composed of cysteine, glutamate, and glycine. Cysteine availability is the main rate-limiting factor in *de novo* biosynthesis of GSH. The system Xc^−^ functions as a cystine/glutamate antiporter in a sodium-independent manner and consists of SLC7A11 (also known as xCT) and SLC3A2. SLC7A11 is the light chain subunit of system Xc^−^ with 12 transmembrane domains, and mediates the activity of system Xc^−^ to specifically transport cystine and glutamate. SLC3A2, the heavy chain component, is a single transmembrane protein that acts as a chaperone protein to regulate the localization and protein stability of SLC7A11 (Nakamura et al., 1999; Shin et al., 2017). In extracellular environment, cysteine is often found in its oxidized form, cystine, and the cystine transported into cells through the system Xc^−^ will be immediately reduced into cysteine to synthesize GSH. Recently, it has been reported that the limiting of exogenous cystine can promote the ferroptosis of pancreatic cancer cells by enhancing the transaminase enzyme GOT1 inhibition (Kremer et al., 2021). The canonical function of GOT1 is to regulate the malate-aspartate shuttle to generate reduced NADPH, which is used to maintain redox balance and proliferation in PDAC. Inhibition of GOT1 impairs mitochondrial oxidative phosphorylation and iron release in PDAC cells, thereby increasing their susceptibility to ferroptosis. In addition, high extracellular glutamate levels could inhibit system Xc^−^ function and cause ferroptosis. Increasing SLC7A11 expression induced by anti-VEGF treatment in glioma leads to the accumulation of extracellular glutamate, which subsequently promotes the proliferation and activation of regulatory T cells (Tregs) in glioblastoma (Long et al., 2020). Like the iron-carrier protein transferrin, the extracellular amino acid glutamine has been discovered as an inducer of ferroptotic cell death (Gao et al., 2015). Furthermore, the glutamine-driven intracellular metabolic process glutaminolysis, which is highly active in cancer cells, is required for ferroptosis triggered by starvation of full amino acids or of cystine alone (Gao et al., 2015).

With the ability to import cysteine from and export glutamate into the extracellular environment, SLC7A11 can exert effects on the interplay with cancer cells and TME, which in turn influence tumor growth and response to cancer therapies. SLC7A11 has been regarded as a primary TME-redox state homeostasis regulator and controls the prooxidant levels including O_2_^•-^ and H_2_O_2_ (Chaiswing and Oberley, 2010). It has been reported that CAFs released cysteine and GSH can protect ovarian cancer cells from chemoresistance-induced oxidative stress, leading to resistance to platinum-based chemotherapy. However, IFNγ produced by effector CD8^+^ T cells downregulates SLC7A11 and SLC3A2 by reducing the release of cysteine and GSH from CAFs, thereby inhibiting cystine uptake in cancer cells, resulting in the induction of tumor lipid peroxidation and ferroptotic cell death (Wang et al., 2016). The role of effector CD8^+^ T cells in abrogating CAFs-mediated chemoresistance indicates the anti-cancer potential of the interplay between chemotherapy and immunotherapy. Meanwhile, Wang et al. further proved that the combination of cyst(e)inase (an engineered enzyme which depletes extracellular cystine and cysteine) with PD-L1 blockade synergistically inhibit cystine uptake through system Xc^−^ in tumor cells, resulting in intracellular GSH deficiency and thus inducing potent tumor cell ferroptosis (Wang et al., 2019). In summary, T cells activated by immune checkpoint inhibitor (ICI) therapy can cause amino acid metabolic reprogramming in tumor cells, ultimately contributing to ICI-induced anti-tumor T cell immunity. In addition, ferroptotic cancer cells can release some signals, notably DAMPs and eicosanoids, which increase their immunogenicity to induce tumor-specific immune responses, enhancing the efficacy of ICI therapy (Turubanova et al., 2019; Yu et al., 2020). Of note, SLC7A11 deficiency has no effect on the anti-tumor activity of T cells, but inhibits mouse T cells proliferate *in vitro* (Arensman et al., 2019). Thus, the role of SLC7A11 in immune cells within the TME warrant further investigation. Taken together, the interaction of amino acid metabolism and immune cells within the TME precisely regulates cancer cell ferroptosis and tumor progression.

### Lipid Metabolism and Ferroptosis Interact to Regulate Tumor Immunity Microenvironment

Lipid metabolism is tightly linked to the cell sensitivity to ferroptosis (Figure 2). Oxidized PUFAs, especially oxidized AA and AdA, have been shown to drive cells to ferroptosis, but exogenous MUFAs, such as exogenous oleic acid (OA) and palmitoleic acid (POA), can protect cancer cells from ferroptosis (Yang et al., 2016; Kagan et al., 2017; Magtanong et al., 2019). Blood and lymphatic vessels are the main sources of intracellular FAs. Meanwhile, the absorption and release of FAs are strictly regulated by the state of the cells and external stimuli. Cancer cells always display aberrant lipid metabolism, which has been regarded as one of the hallmarks of aberrant cell growth and cancer progression. Elevated *de novo* synthesized lipids and FA oxidation are necessary for cancer cells to produce energy and required for post-translational modification of proteins (Carracedo et al., 2013; Rohrig and Schulze, 2016). The intricate interactions within the TME and adjacent stroma frequently impact the ways how cancer cells utilize lipids. For example, the presence of tumor low oxygen (hypoxia) environment results in the stabilization and activation of hypoxia inducible factors (HIFs), which regulate the transcription of target genes and consequently modulate lipid metabolism (Metallo et al., 2011). Activation of HIF1 in the head and neck tumor cells enhances SIAH2-mediated OGDN2 degradation, resulting in glutamine-derived carbon shunting to FA synthesis (Sun and Denko, 2014). The sterol regulatory element binding proteins (SREBPs), which control cholesterol and FA synthesis and uptake, are upregulated in hypoxic conditions and essential for glioblastoma multiforme cell survival (Lewis et al., 2015). SREBP can control the FA desaturases to introduce the double bonds into newly synthesized FAs and protect cancer cells from lipotoxicity under nutrient stress (Peck and Schulze, 2016). Furthermore, in response to metabolic stress and extracellular acidification, SREBP2 drives acetyl-CoA synthetase 2 (ACSS2) expression enabling the conversion of acetate to acetyl-CoA for lipid synthesis to promote tumor progression (Schug et al., 2015; Kondo et al., 2017). Recently evidence suggests that CAFs may also influence lipid transfer and uptake from the TME by enhancing the expression of FATP1 in breast cancer (Lopes-Coelho et al., 2018). In summary, the TME regulates fatty acid pools in cancer cells, which in turn affects ferroptosis sensitivity.

On the other hand, FAs secreted by cancer cells into the microenvironment could also impact infiltrating immune cell function and phenotype during tumor progression. During the stromal invasion, cancer cells can be impacted by circulating free fatty acids (FFAs) and other lipid molecules, which can substantially affect cell signaling or supply new substrates for cell development. Cancer cell-secreted FAs within the TME may stimulate TAMs to an M2 phenotype, which is marked by an increase in fatty acid oxidation (FAO) (Cook and Hagemann, 2013). The role of FFAs from circulation or within the TME in modulating the CD8^+^ T cell effector functions depends on context. Within hypoxic environments, Tregs in glioblastoma utilize extracellular FFAs to support the suppression of CD8^+^ T cells (Miska et al., 2019). Accumulation of lipid within lipid droplets in tumor-associated DCs reduces the antigen presentation, leading to dysfunction of DCs and poor stimulation of T cell responses, thereby modulating anti-tumor immunity (Herber et al., 2010; Ramakrishnan et al., 2014). Several studies demonstrate that exogenous lipids can disrupt the mTORC1-mediated glycolytic increase, which is necessary for the synthesis of granzyme B and IFNγ, leading to a deleterious impact on melanoma natural killer (NK) cell effector functions and their capacity to respond to stimuli, especially in the context of obesity (Michelet et al., 2018). Moreover, when the availability of glucose in the TME is limited, neutrophils and polymorphonuclear myeloid-derived suppressor cells (PMN-MDSCs) can utilize FAO to support ROS production and lead to T cell suppression (Hossain et al., 2015; Rice et al., 2018). Therefore, exogenous lipids within the TME can influence the activity of immune cells to promote tumor progression.

As previously stated, lipid peroxidation is a hallmark of ferroptosis and is tightly linked to the process of lipid metabolism. The interplay between ferroptosis and lipid metabolism is important in anti-tumor immunity modulation. Accumulating evidence has revealed that ferroptotic cancer cells can affect the activity of immune cells by releasing various oxidized lipid metabolites. In ferroptotic cancer cells, AA can be metabolized to different eicosanoids and their derivatives, such as prostaglandin E2 (PGE2), 15-hydroperoxy-eicosatetra-enoyl-phosphatidylethanolamine (15-HpETE-PE), 15-HETE, 12-HETE, and 5-HETE, through the function of various pathways and released into the tumor immune microenvironment (Friedmann Angeli et al., 2019). PGE2 has been identified as an immunosuppressive factor that suppresses the anti-tumor functions of NK cells, conventional type 1 dendritic cells (cDC1s), and cytotoxic T cells to alter the TME and promote colon cancer cell proliferation, migration and invasion (Johnson et al., 2020). The pro-ferroptotic lipid 15-HpETE-PE that released by ferroptotic cancer cells will induces immune cells undergo ferroptosis in the TME (Kapralov et al., 2020). Taken together, lipid metabolic abnormalities of ferroptotic cancer cells can impact infiltrating immune cell function and phenotype in the TME, while TME often affects the way cancer cells utilize lipids. The interaction of lipid metabolism and immune cells within the TME builds a regulatory network for modulating ferroptosis sensitivity of cancer cells.

### The Role of Lactate Metabolism in Ferroptosis Within the TME

**FIGURE 3:**
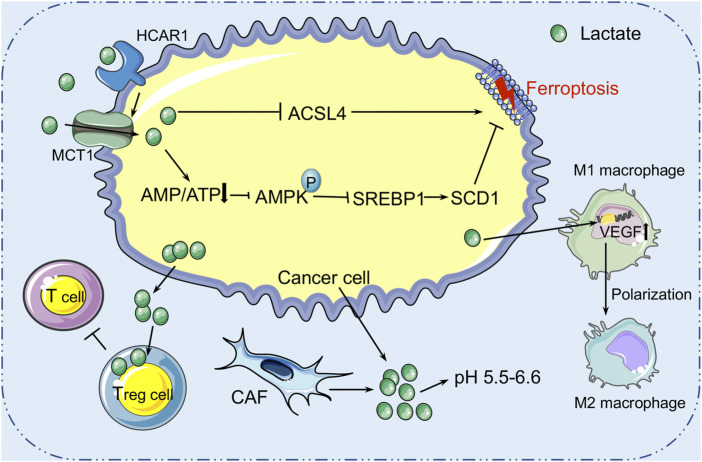
The role of lactate metabolism in ferroptosis within the TME. Lactic acid is mainly derived from tumor cells and cancer-associated fibroblasts (CAFs) in the tumor microenvironment. High concentration of lactic acid forms an acidic environment with pH 5.5–6.6 in the TME. Cancer cells secrete lactic acid to nourish Treg cells to protect the tumor from immune attack. Lactic acid can also induce the formation of mono-unsaturated fatty acids through the HCAR1/MCT1-SREBP1-SCD1 pathway, which contributes to the resistance of liver cancer cells to ferroptosis induced by oxidative stress. HCAR1, hydroxycarboxylic acid receptor 1; MCT1, monocarboxylate transporter-1; SREBP1, sterol regulatory element-binding protein-1; SCD1, stearoyl-CoA desaturase-1.

Lactate is a hydroxycarboxylic acid that is used as a fuel source by the heart, brain, and skeletal muscle under physiological conditions (Gladden, 2004). Based on the “lactate shuttle theory,” lactate can transcend compartment barriers and shuttle occur within and among cells, tissues, and organs to deliver oxidative and gluconeogenic substrates as well as in cell signaling (Brooks, 2009; 2018). In 1926, *Otto Warburg* demonstrated that glucose, as a primary energy source, was heavily ingested in several cancer cells, producing an excessive amount of lactate, even in the presence of oxygen (Warburg et al., 1927). Tumor cells and CAFs in the TME are the main sources of lactate production. Due to the accumulation of extracellular lactate, the extracellular pH is ranging between 5.5 and 6.6 (Webb et al., 2011; Justus et al., 2013). It seems that the acidic environment in tumors is associated with the more aggressive tumor characteristics such as metastasis, angiogenesis, and therapy resistance (Estrella et al., 2013; Garcia-Canaveras et al., 2019). For instance, lactate induces the expression of transforming growth factor-β2 (TGF-β2), a key regulator of tumor cell invasion, in glioma cells (Baumann et al., 2009). Therefore, increasing evidence reveals that lactate is not only a major energy source (Brooks et al., 1991; Bergman et al., 1999) but also a signaling molecule (Brooks, 2002; Hashimoto et al., 2007; Brooks, 2009) that has many regulatory roles in the TME, and particular relevance to oxidative stress resistance (Tauffenberger et al., 2019; Tasdogan et al., 2020) and lipid biosynthesis (Chen et al., 2016). Pucino et al. reported that the accumulation of extracellular lactate contributes to regulating the production of various lipid substances, including acetyl-CoA and citrate, *via* AMP-activated protein kinase (AMPK) or signal transducer and activator of transcription 3 (STAT3) signaling (Pucino et al., 2019). These finds suggest that lactate may play a role in cancer cell ferroptosis regulation. Recently, Zhao et al. discovered the aberrantly high level of lactate in the HCC extracellular environment could promote ATP production and further deactivate the AMPK signals (Zhao et al., 2020). In addition, it was also confirmed that lactate can induce the formation of MUFAs through the HCAR1/MCT1-SREBP1-SCD1 pathway, which contributes to the resistance of liver cancer cells to ferroptosis induced by oxidative stress (Zhao et al., 2020) (Figure 3). Therefore, the role of lactate receptor HCAR1 and transporter MCT1 in the maintenance of redox homeostasis has been found, suggesting that HCAR1 may be a potential therapeutic target for ferroptosis in HCC (Zhao et al., 2020). In addition, lactate in the TME can impair the immune system and promote tumor development by triggering and recruiting immunosuppressive related cells and molecules (de la Cruz-Lopez et al., 2019; Ivashkiv, 2020). It has been reported that Treg cells can use lactate to maintain the strong suppression of effector T cells within the TME (Watson et al., 2021). Tumor cell-derived lactate can polarize TAM into M2 type, activate the expression of VEGF and arginase 1 (ARG1) through the HIF1-α signaling pathway in macrophages, and assist TAM to promote tumor growth (de la Cruz-López et al., 2019; Zhang et al., 2020). Overall, high lactate concentration promotes acidification of the TME and suppresses the immune system, thereby contributing to immune evasion and tumor growth, invasion, and migration. The definitive information regarding the lactate in regulating ferroptosis of cancer cells is limited. Considering the role of lactate in immune suppression, the hypothesis that lactate modulates the sensitivity to ferroptosis through the immune system within the TME deserves consideration in future studies.

## Ferroptosis-based Crosstalk Between Tumor Cells and Immune Cells in the TME

**FIGURE 4:**
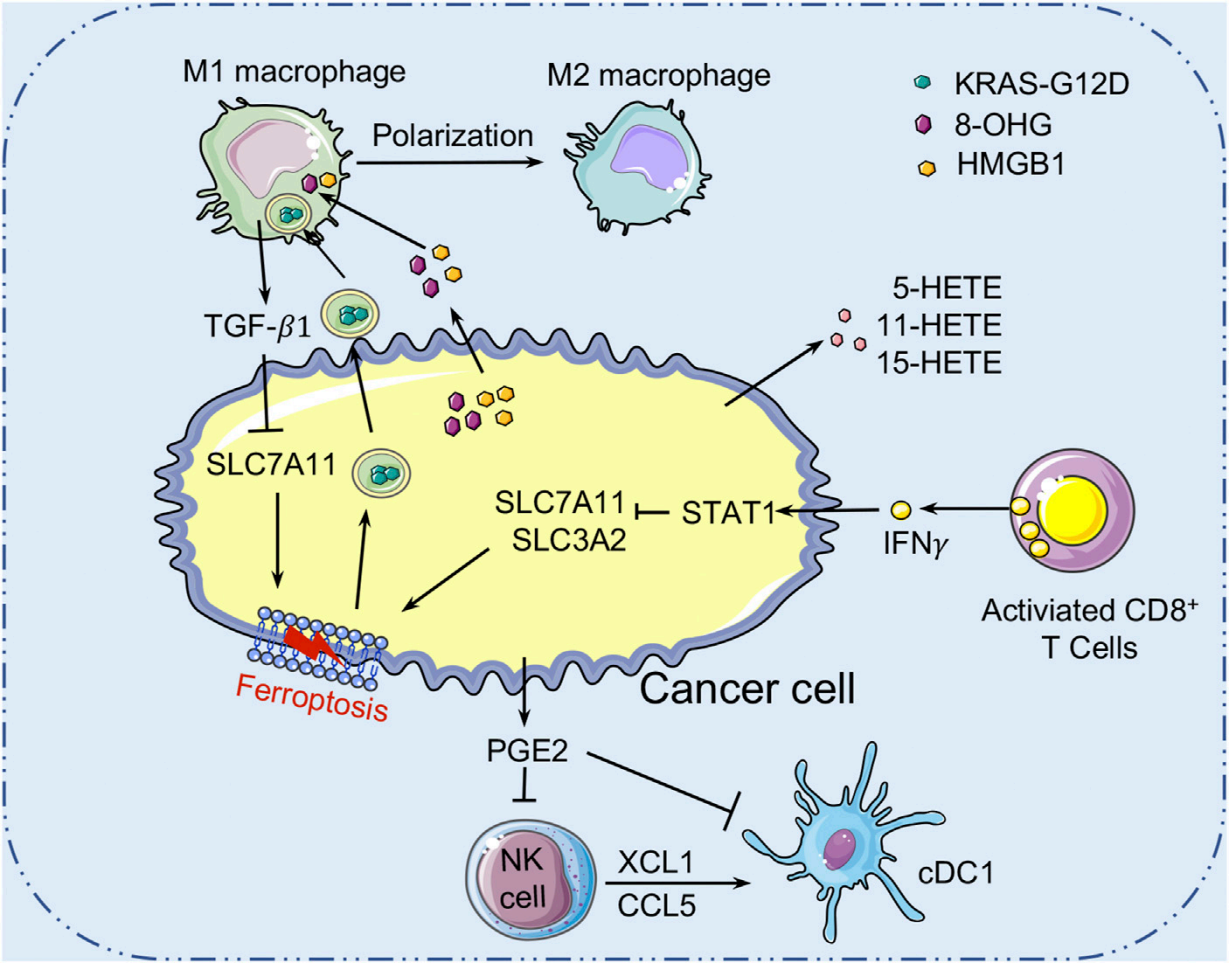
Crosstalk between ferroptosis and tumor immunity. Ferroptotic cancer cells release the KRAS-G12D proteins. Extracellular KRAS-G12D proteins are packaged into exosomes, which are absorbed by macrophages through an AGER-dependent mechanism, thereby inducing macrophages to switch to the M2 phenotype to accelerate cancer development. Ferroptotic cancer cells can also release HMGB1 and 8-OHG to affect the innate immune cells’ functions in the TME. In addition, ferroptotic cancer cells released PGE2 can inhibit the infiltration of cDC1s by suppressing the chemokines CCL5 and XCL1 that secreted by NK cells. PGE2 can also downregulate chemokine receptors to block cDC1s directly. Many cell types (such as macrophages) release TGF-β1 to activate SMAD proteins to inhibit the expression of SLC7A11, thereby promoting the ferroptosis of cancer cells. AGER, advanced glycosylation end-product-specific receptor; HMGB1, high mobility group box1; 8-OHG, 8-Hydroxyguanosine; cDC1s, conventional type 1 dendritic cells; PGE2, Prostaglandin E2; TGF-β1, transforming growth factor-β1.

Mounting evidence suggests that ferroptosis is tightly linked to tumor suppression and immunity (Figure 4). The role of ferroptosis in tumor immunity is determined by the interactions between cancer cells and distinct immune cell subgroups. As all we known, ferroptosis is an iron-dependent form of RCD caused by lipid peroxidation and plays an important role in cancers. Many metabolites, particularly those involved in the metabolism of iron, lipids, amino acids and lactate, regulate the complicated ferroptotic response by directly or indirectly regulating iron accumulation or lipid peroxidation in cancer cells. For example, AA and AdA, the metabolites of lipid metabolism, can be esterified by ACSL4 and LPCAT3 into PE, while LOXs may oxidize AA-PE and AdA-PE to AA-PE-OOH and AdA-OOH (e.g., 5-HETE, 11-HETE, and 15-HETE), which leads to membrane rupture and increases membrane permeability, leading to ferroptosis. Recently, several researches have indicated that ferroptotic cancer cells may release a variety signals to activate immune cells (e.g., neutrophils) in the TME (Friedmann Angeli et al., 2019). DAMPs, which are mainly released by dead or dying cells, are thought to be mediators of inflammation and immune responses in various types of RCD. Extracellular DAMPs can recruit and activate effector immune cells by binding to their receptors on various immune cells, such as macrophages and monocytes, aggravating inflammatory responses that support tumor growth (Tang et al., 2012). DAMP release, on the other hand, can also mediate immunogenic cell death, which can enhance anti-tumor immunity (Galluzzi et al., 2017). Ferroptotic cancer cells can release and activate distinct signals including DAMPs (e.g., HMGB1, KRAS-G12D, and 8-OHG) or oxidized lipid mediators (e.g., 4HNE, oxPLs, LTB4, LTC4, LTD4, and PGE2) that may modulate inflammatory and anti-tumor immunity. HMGB1 released by ferroptotic cancer cells is a prototypical DAMP involved in the immunogenicity of cancer cells, and it triggers an inflammatory response in macrophages through binding to advanced glycosylation end-product-specific receptor (AGER/RAGE) (Wen et al., 2019). Interaction of RAGE and HMGB1 leads to the phosphorylation of MAPKs and activation of the NF-κB signaling pathway in macrophages, which stimulates the release of pro-inflammatory cytokines (e.g., TNF-α and IL-1) by macrophages to promote tumor progression (Wen et al., 2019). Meanwhile, the ferroptosis-mediated DAMP release may promote tumor progression by driving the polarization of macrophages in TME. In this context, KRAS-G12D can be released within exosomes by ferroptotic cancer cells and taken up by macrophages through an AGER-dependent mechanism, resulting in the polarization of macrophages to an M2 phenotype and promotion of tumor growth (Dai et al., 2020a). Moreover, it has been reported that the PGE2 released by ferroptotic cancer cells could disrupt the anti-tumor immunity of the innate immune system by targeting NK cells and cDC1s, thereby promoting the progression of colon cancer (Bottcher et al., 2018). However, reports on the roles of these lipid-derived products released by ferroptotic cancer cells are still rare and require further investigation. In conclusion, the DAMPs and lipid mediators released by cancer cells might form a complicated network that modulates anti-tumor immunity during ferroptosis. At the same time, further research is warranted to figure out the immunomodulatory function of ferroptotic cancer cells in anti-tumor immunity.

Recent studies have found that CD8^+^ T cells can trigger ferroptosis in cancer cells, indicating that ferroptosis might be modulated by immune cells (Figure 4). Mechanistically, CD8^+^ T cell-derived IFNγ inhibits SLC7A11 expression in cancer cells through activating the transducer and activator of transcription 1 (STAT1), thereby inducing tumor cell ferroptosis and contributes to the anti-tumor efficacy of immunotherapy (Wang et al., 2019). TGF-β1 released by macrophages can promote cancer cells ferroptosis by activation of the SMAD-related signaling to inhibit the expression of SLC7A11 (Kim et al., 2020). In addition, the lymphatic system promotes tumor metastasis by increasing the production of ACSL3-dependent MUFAs that protects melanoma cells from ferroptosis (Chen et al., 2021). Moreover, immune cells in the TME also influence a variety of metabolisms, including the metabolism of iron, lipid, and lactate, to modulate the ferroptosis sensitivity of cancer cells. The interactions between ferroptosis and the immune microenvironment in cancer cells provide a novel way to cancer therapies.

## Role of the Tumor Microenvironment in Ferroptosis-dependent Cancer Therapy

Cancer therapies that are widely utilized include surgery, chemotherapy, hormone therapy, radiation therapy, and immunotherapy. The purpose of these strategies is to remove or kill cancer cells. According to recent studies, the complicated role of ferroptosis in tumor therapy and tumorigenesis is influenced not only by the tumor-associated genes, but also by the TME. The novel chemotherapy, immunotherapy, and radiation therapy targeting ferroptosis-related pathways and metabolism have shown promising potential for cancer therapies.

### Immunotherapy

Recently, Wang et al. reported that ferroptosis induction-based treatment can be combined with ICIs to enhance the potency of killing cancer cells (Wang et al., 2019). ICIs (e.g., anti-PD-1 and anti-PD-L1 antibodies) can activate cytotoxic T cells to exert anti-tumor immunity, thereby inducing ferroptosis in cancer cells. It has been reported that ICI anti-PD-L1 antibodies can potentiate ferroptosis activators (e.g., erastin and RSL3) induced tumor growth inhibition *in vitro* and *in vivo* (Wang et al., 2019). Specifically, anti-PD-L1 antibodies stimulate CD8^+^ T cells to release cytokine IFNγ to activate the JAK-STAT1 pathway in cancer cells, thereby reducing the expressions of SLC7A11 and SLC3A2, and ultimately increasing the sensitivity of cancer cells to ferroptosis (Wang et al., 2019). In melanoma patients, an increase in ICIs efficacy is consistently associated with a decrease in SLC3A2 expression (Wang et al., 2019). Furthermore, depletion of STAT1 in tumor cells disrupts the IFNγ-mediated SLC7A11 suppression and reverses RSL3-induced ferroptosis. Notably, STAT1 can be activated by a variety of ligands, which means that other cytokines may have similar effects to that of IFNγ in triggering ferroptosis. Meanwhile, the released DAMPs play a dual role in anti-tumor immunity. As mentioned above, ferroptotic cancer cells release HMGB1 to promote the inflammatory responses of macrophages through binding to AGER (Wen et al., 2019). Therefore, blocking the HMGB1-AGER pathway with small molecules or genetic methods could limit the inflammatory response mediated by ferroptosis (Chen et al., 2021). These DAMPs, oxidized lipid mediators, and cytokines released into the extracellular formed a complex regulatory network between ferroptotic cancer cells and the immune system. As a result, immunotherapy combined with ferroptosis induction is a promising method that synergistically promotes anti-tumor effect.

### Radiotherapy

Radiotherapy can directly cause oxidative damage to lipid membrane, leading to the accumulation of toxic lipid peroxidation and triggering ferroptosis in cancer cells. Meanwhile, recent studies suggest that radiotherapy can enhance the anti-tumor effect by synergizing with ferroptosis triggers and immunotherapy. Mechanically, radiotherapy can activate Ataxia-Telangiectasia mutated gene (ATM) to produce IFNγ and synergizes with immunotherapy-activated CD8^+^ T cells to suppress SLC7A11 expression in cancer cells. In addition, radiotherapy also upregulates ACSL4 expression in cancer cells, thereby increasing lipid peroxidation and inducing ferroptosis (Lei et al., 2020). Li et al. reported that mitochondrial DNA damage can activate the STING1/TMEM173-dependent DNA sensing pathway and lead to ferroptotic cancer cell death (Li C. et al., 2021). Similarly, this or other DNA sensor pathways activated by radiotherapy may similarly enhance the anti-tumor effect of inducing ferroptosis. Thus, the combination of radiotherapy and immunotherapy also increases the sensitivity of cancer cells to ferroptosis, indicating that inducing ferroptosis is a promising anti-cancer strategy.

### Chemotherapy

Nanoparticle-based delivery of ferroptosis inducers can precisely and efficiently kill tumor cells with low toxicity by controlling the release of the drug and improving the pharmacokinetic properties of drugs. For example, due to the poor solubility of ferroptosis inducer withaferin A in water, withaferin A can be formulated in the amphiphilic degradable pH-sensitive nanocarrier to improve the convenience of drug delivery and reduce drug toxicity (Hassannia et al., 2018). Furthermore, nanoparticle-based ferroptosis induction is often combined with other treatments to enhance the anti-tumor effect. Chemodynamic therapy (CDT) is one of the ROS-dependent anti-cancer therapies that generate ROS through the iron-mediated Fenton reaction to kill cancer cells efficiently (Tang et al., 2019). Khalaf et al. used the novel tumor-targeted conjugated polymer nanoparticles (CPNPs) to deliver iron to the targeted tumor cells to trigger ferroptosis-assisted CDT through Fenton reaction that produces ROS (Jasim and Gesquiere, 2019). They conjugated iron-carrying CPNPs with the endothelin 3 (EDN3-CPNP), which specifically targets the melanoma endothelin-B receptor (EDNRB), and showed enhanced melanoma tumor targeting and tumor cell killing effects (Jasim and Gesquiere, 2019). In addition, nanoparticle-based ferroptosis induction can combination with photodynamic therapy (PDT) to markedly enhance the anti-tumor effect of PDT (Zhu et al., 2019). In order to counteract the depressant effect of the hypoxic TME in solid tumors on PDT, researchers constructed a nanoparticle containing the photosensitizer chlorin e6 (Ce6) and the ferroptosis inducer erastin *via* supramolecular interaction (Zhu et al., 2019). The obtained Ce6-erastin nanodrugs by cancer cells subsequently trigger ferroptosis and increases oxygen concentration, thus promoting the PDT anti-tumor efficacy upon light irradiation (Zhu et al., 2019). It has been reported that nanoparticle-based ferroptosis induction can also collaborate with photothermal therapy (PTT) to construct the nanoparticles named SRF@MPDA-SPIO. This nanoparticle is composed of ferroptosis inducer sorafenib (SRF) and super-paramagnetic iron oxide (SPIO) loaded into the mesopores or onto the surface of mesoporous polydopamine (MPDA) NPs through π–π stacking and/or hydrophobic interaction. In this system, the PTT (provide by MPDA NPs under laser emissions) synergizes with ferroptosis (induced by SPIO and SRF) to develop a promising cancer therapy (Guan et al., 2020). For chemotherapy, the ability of nanoparticle-induced ferroptotic cell death is highly dependent on nanoparticle structure and chemical modifications.

## Conclusions and Perspectives

Over the last few years, we have gained a better understanding of ferroptosis or ferroptosis-like cell death. In general, the sensitivity of cancer cells to ferroptosis is dependent on the state of cellular metabolisms, such as the metabolisms of lipid, iron, lactate, and amino acid. A variety of reagents, most of which are used in cancer treatment, have recently been found with the ability to modulate the ferroptotic process by directly or indirectly targeting iron metabolism and lipid peroxidation. Although its involvement in tumor treatment is currently unknown, pharmacologically triggering ferroptosis is a promising therapeutic therapy. Of note, hypoxic environment and infiltrating immune cells within the TME influence cancer cells’ vulnerability to ferroptosis by altering the metabolism of cancer cells. In turn, molecular factors involved in the metabolic pathways and released by ferroptotic cancer cells can affect infiltrating immune cell function and phenotype and reshape the tumor niche.

In carcinogenesis, these secreted DAMPs within the TME induced by ferroptotic damage exhibit a dual function in tumor growth and inhibition. However, the function and phenotype changes of infiltrating immune cells in response to ferroptosis remain unclear. Therefore, it is necessary to conduct more detailed research on the effect of ferroptosis in the tumor immune microenvironment to provide a new approach for cancer treatment through ferroptosis-induced cell death and anti-tumor immunity. Furthermore, the mechanism of TME in regulating the ferroptotic process (especially the metabolism of lipid, iron, amino acid, and lactate) of cancer cells still needs to be clarified. In summary, a better understanding of the TME in regulating ferroptosis will create a new way to develop novel cancer-eradication therapies.
